# Revision of the genus *Gryposmylus* Krüger, 1913 (Neuroptera, Osmylidae) with a remarkable example of convergence in wing disruptive patterning

**DOI:** 10.3897/zookeys.617.10165

**Published:** 2016-09-15

**Authors:** Shaun L. Winterton, Yongjie Wang

**Affiliations:** 1 California State Collection of Arthropods, California Department of Food & Agriculture, Sacramento, California, USA; 2 College of Life Sciences, Capital Normal University, Beijing, China

**Keywords:** Osmylidae, convergence, camouflage, lacewing

## Abstract

The charismatic lance lacewing genus *Gryposmylus* Krüger, 1913 (Osmylidae: Protosmylinae) from South East Asia is revised with a new species (*Gryposmylus pennyi* **sp. n.**) described from Malaysia. The genus is diagnosed and both species in the genus redescribed and figured. An extraordinary example of morphological convergence is presented, with disruptive camouflaging wing markings in *Gryposmylus pennyi* **sp. n.** being remarkably similar to the South American green lacewing *Vieira leschenaulti* Navás (Chrysopidae).

## Introduction

Lance lacewings (Osmylidae) are a charismatic family of Neuroptera found throughout the world except North America. The family currently contains almost 300 species, both extant and extinct (Oswald 2016). Osmylids are relatively basal representatives of Neuroptera, and are closely related to families such as Nevrorthidae and Sisyridae, as well as Coniopterygidae and Dilaridae (Winterton et al. 2010; Wang et al. 2016). The major works on the family were by Krüger (1912–1915b) early during the previous century, where he erected numerous genera often based on spurious wing venation features; today only approximately 25 genera are considered valid (Kimmins 1940; New 1986; Wang and Liu 2009). The division of the extant representatives of the family into eight subfamilies, including Eidoporisminae, Gumillinae, Kempyninae, Osmylinae, Porisminae, Protosmylinae, Spilosmylinae and Stenosmylinae, is far more stable, with each subfamily relatively easily diagnosable (Krüger 1912–1915b; New 1989). The extinct subfamily (Mesosmylininae) comprising at least seven genera is known from Late Triassic to Mid Cretaceous aged deposits (e.g., Jepson et al. 2009, 2012; Khramov 2014), while another extinct subfamily Cratosmylinae, containing the single species *Cratosmylus* Myskowiak, is known from Brazilian Cretaceous aged deposits (Myskowiak et al. 2015). Finally, the extinct subfamily, Epiosmylinae, is considered a junior synonym of Gumillinae (Lambkin 1988; Wang et al. 2009; Khramov 2014). The putative sister family to Osmylidae is Archaeosmylidae (Late Permian to Early Triassic) and is differentiated from the former based on several wing venation features, such as a non-pectinately branched forewing CuP (see Khramov 2014).

The osmylid subfamily Protosmylinae comprises four extant genera and at least four extinct genera and shares a close relationship with the subfamilies Spilosmylinae (Wang et al. 2010) and Gumillinae. The close relationship between these subfamilies is exhibited by similarities in the wing venation, most notably with the hind wing vein CuP being unbranched, while in other subfamilies the vein is highly pectinately branched along the posterior wing margin. Spilosmylinae and Protosmylinae share a series of features in the male genitalia, including a narrowly arched and apilose gonarcus, parameres present and fused apically into an arch-like shape (subarcus *sensu* Tjeder 1957) in most genera, narrow entoprocesses, abdominal scent glands absent and tergites 8 and 9 separate. In the female genitalia, both subfamilies share the reduction in size of sternite 8 with a concomitant migration of the sclerite posteriorly. During copulation, sternite 8 acts against a depression in the intersegmental membrane immediately posterior to sternite 7. In other subfamilies sternite 8 is regularly shaped and positioned immediately posterior to sternite 7, and is acted upon during copulation by gonopophyses 9.

The Oriental genera *Heterosmylus* Krüger and *Gryposmylus* Krüger are placed in Protosmylinae, along with the monotypic genus *Paryphosmylus* Krüger from Ecuador (Krüger 1912–1915b; Wang et al. 2010). Recent studies using molecular and morphological data also place the Oriental genus *Lysmus* Navás in the subfamily (SLW unpublished data). Fossil protosmylines are known from the Tertiary, namely *Protosmylus* Krüger (Hagen in Berentdi 1856) from Baltic amber and *Osmylidia* Cockerell from Florissant shale deposits (Cockerell 1908; Carpenter 1943). Older fossils are known also from the Mid Jurassic (e.g., *Juraheterosmylus* Wang et al.) (Wang et al. 2010), Late Jurassic (e.g., *Jurosmylus* Makarkin & Archibald) (Khramov 2014b) and Early Cretaceous (e.g., *Protosmylina* Jepson et al.) (Jepson et al. 2009). Clearly, while Protosmylinae are presently distributed only in the Oriental and Neotropical regions, the subfamily has had a much broader distribution previously, with fossils present throughout the Holarctic region.

**Figure 1.**
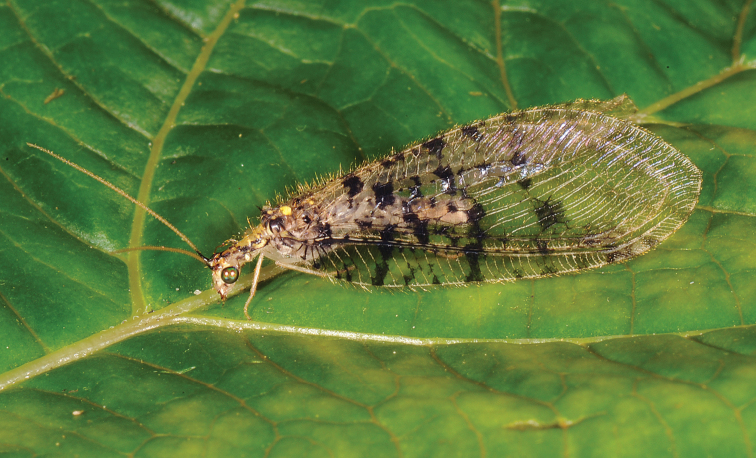
*Gryposmylus pubicosta* (Walker), male (dark form) Sabah, Malaysia (photograph credit: Stephen D. Gaimari).

**Figure 2.**
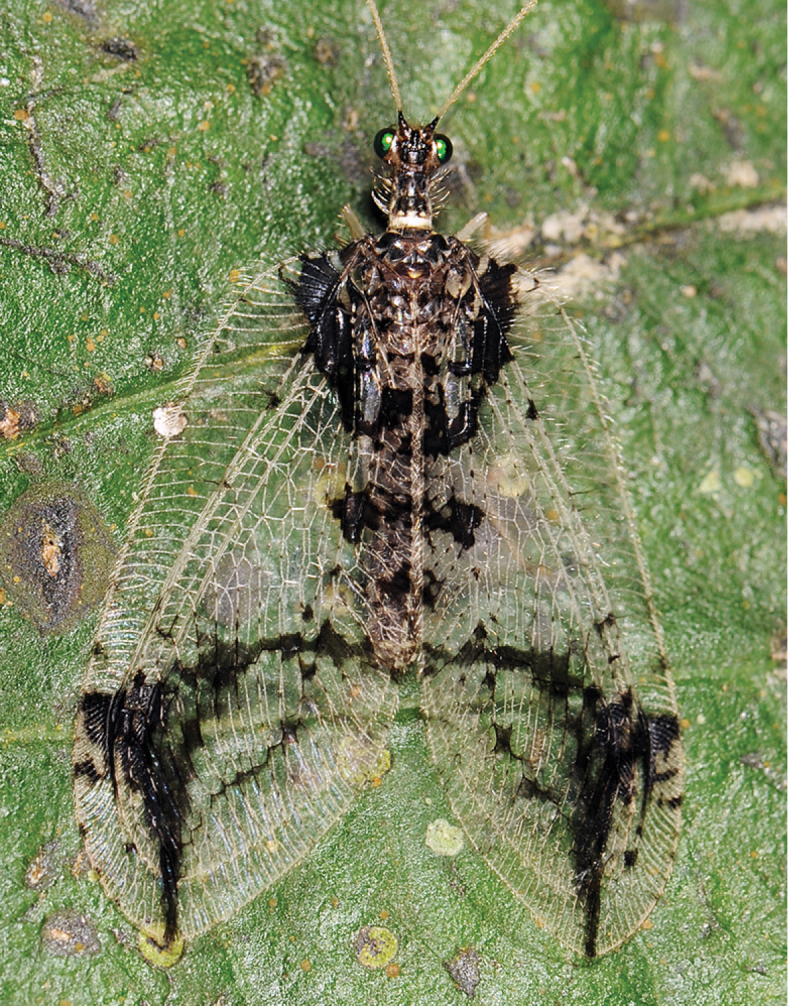
*Gryposmylus pennyi* sp. n., male, Sabah, Malaysia (photograph credit: Stephen D. Gaimari).

Herein we revise the genus *Gryposmylus*, including a redescription of the type species (*Gryposmylus pubicosta* (Walker)) (Fig. 1) and a description a new species (*Gryposmylus pennyi* sp. n.) (Fig. 2). A key to species is provided and both species are figured. The new species of *Gryposmylus* presents a remarkable example of convergence in disruptive camouflage markings in the wings, exhibiting an amazing similarity with a very distantly related green lacewing (Chrysopidae) from South America.

## Materials and methods

Terminology follows Tjeder (1957), Adams (1969) and Wang et al. (2011) with the following modifications based of detailed examinations and a recent assessment of structural homology across all genera of Osmylidae and related families of Neuroptera (SLW, unpublished data). Figure 5 depicts the various male genitalic structures (colour-coded) typically exhibited in Protosmylinae. In the male genitalia the baculum (*sensu* Tjeder 1957) is the anterior arm of the gonarcus (red colour). This structure is present in some subfamilies and may be articulated (e.g., Spilosmylinae) or fused with the rest of the gonarcus (e.g., Kempyninae, Protosmylinae). The parameres (green colour) herein are equivalent to the subarcus of Tjeder (1957). Parameres are present only in Osmylinae (paired structures), Spilosmylinae (fused ventrally and U-shaped) and Protosmylinae (fused dorsally and arch-shaped). The parameres are always closely associated with the mediuncus (pink colour), either immediately flanking the mediuncus (e.g., Osmylinae), cradling from below (e.g., Spilosmylinae) or immediately anterior to it (Protosmylinae). We follow Adams (1969) in the use of mediuncus for the main intromittent organ, which is equivalent to the parameres of some authors (e.g., Tjeder 1957; New 1986), or more recently the gonocoxite 10 complex of Aspöck and Aspöck (2008) and Martins et al. (2016), or gonocoxites by Ardila-Camacho and Noriega (2014). The gonarcus is variable in form depending on the subfamily, being relatively narrow, arching medially and lacking setae in Protosmylinae and Spilosmylinae. In Porisminae, Stenosmylinae, Kempyninae, Eidoporisminae and especially Osmylinae, the gonarcus is large and observable externally, with distinct setal pile evident. Entoprocesses (blue colour) are fused laterally on the gonarcus (*sensu* Tjeder 1957), and are equivalent to gonocoxite 9 of Adams (1969). In Spilosmylinae and Protosmylinae they are narrow and elongate, while in Osmylinae they are more reduced to shorter and broader processes. In Kempyninae and Stenosmylinae they are larger and subtriangular in shape. Aspöck and Aspöck (2008) and Martins et al. (2016) interpreted the gonarcus and entoprocessus as a complex of gonocoxite 9 and gonopophyses 9, respectively. In the female genitalia (Fig. 6), the structure of the female sclerites is also variable among subfamilies. The enlarged gonocoxite 9 (=gonopophyses lateralis) is closely associated anteriorly with gonopophysis 9 (=sternite 9 of Wang et al. (2011)) in Protosmylinae, Spilosmylinae and Osmylinae and two separate sclerites are clearly visible. By contrast, in more derived subfamilies Porisminae, Kempyninae, Eidoporisminae and Stenosmylinae, gonopophyses 9 is not closely associated as a regular sclerite with gonocoxite 9, but instead is more elaborately shaped as a single articulating sclerite which acts upon sternite 8. In all subfamilies there is a terminal stylus (gonostylus 9). Sternite 8 (*sensu* New 1986) has also been referred to as the subgenitale (*sensu* Tjeder 1957), or as a fusion of gonocoxite8+gonopophyses 8 by Aspöck and Aspöck (2008) and Martins et al. (2016). The shape and position of this sclerite is highly variable. In Osmylinae, Porisminae, Kempyninae, Eidoporisminae and Stenosmylinae it is a plate-like sclerite immediately posterior to sternite 7. In Kempyninae and Stenosmylinae it is frequently modified into a bowl-like structure. In Protosmylinae and Spilosmylinae, sternite 8 is much smaller in size and located posteriorly as a small knob-like process.

Wing vein terminology used here follows Makarkin et al. (2011) with regard to the identity of vein MA in both wings. Based on recent unpublished studies on wing venation and tracheation (SLW unpublished data) we disregard the assumption that the MA vein is fused anteriorly with R and that it is represented in both wings as the posterior-most vein of the Rs field (*sensu* Kukalová-Peck and Lawrence 2004) (Fig. 4). Consequently, we do not consider the sigmoid vein as the remnant of vein MA.

Genitalia were macerated in 10% KOH to remove soft tissue, then rinsed in distilled water and dilute glacial acetic acid, dissected in 80% ethanol and subsequently stained with a solution of Chlorazol Black in 40% ethanol. The dissected genitalia were placed in glycerine in a genitalia vial mounted on the pin beneath the specimen.

## Taxonomy

### Gryposmylus

Taxon classificationAnimaliaNeuropteraOsmylidae

Krüger, 1913: 32.

Figs 1 , 2 , 3 , 4

#### Type species.

*Chrysopa pubicosta* Walker 1860: 183, original designation.

#### Diagnosis.

Forewing length 15–18 mm. Antennae much shorter than forewing length; head with posterior genal area relatively wide; prothorax length subequal to width; female forecoxa lacking pedicellate setae or anterior processes; wing ovoid, not falcate along posterior margin; costal area broad basally with basal crossveins arranged radially, costal crossveins simple with occasional forking; interlinking crossveins absent from entire wing margin; wing venation with relatively few crossveins; two gradate series well defined, divergent in orientation; single basal subcostal crossvein present; forewing with seven branches of Rs present, basal branch of Rs diverging close to origin of Rs on R_1_; forewing M vein branching in proximal half of wing, basal to origin of basal branch of Rs; hind wing CuP as a single vein branching just before wing margin; male genitalia with gonarcus narrowly arched medially and apilose, anterior arms of gonarcus (=baculum) present, non-articulated; parameres present, ends fused anteriorly and forming an arch-shape; entoprocesses narrow, spatulate distally and curved dorsally; male tergites 8 and 9 as separate sclerites, scent glands absent; female genitalia with sternite 8 positioned posteriorly, small and knob-like (=subgenitale), hollowed depression in the membrane immediately posterior to sternite 7; sternite 7 unmodified; spermatheca as single lobe, folded onto itself; spermathecal duct greatly elongate and coiled around spermatheca.

#### Included species.

*Gryposmylus pubicosta* (Walker); *Gryposmylus pennyi* sp. n.

#### Comments.

*Gryposmylus* is a distinctive Oriental genus that is the putative sister genus of *Lysmus*. Both genera have the basal branch of forewing vein Rs diverging close to the origin of Rs on R. In *Gryposmylus* forewing vein M forks basally, or equal with, the origin of the basal branch of Rs, while in *Lysmus* this fork is distal to the origin of the basal Rs branch. The costal margin of *Gryposmylus* is wider basally than in all other Protosmylinae genera, and the basal 7–8 costal crossveins are arranged in a slight radiating pattern, while in other genera they are usually parallel, or only the basal 2-3 veins are radially oriented. Also in *Gryposmylus* the forewing gradate series is generally divergent in orientation, while in other Protosmylinae genera they are subparallel. There is some variation among individuals in both *Gryposmylus* and *Lysmus* and the distinction of the genera is not consistently defined. At this time we maintain them as separate genera until more species are known and the limits of this variation are known.

#### Key to species of *Gryposmylus*

**Table:** 

1	Head, thorax and abdomen almost entirely black with brown mottled patterning; forewing extensively marked along posterior margin and distally, distinct dark streak apically in both wings, pterostigmal marking relatively large (Fig. 3B)	***Gryposmylus pennyi* sp. n.**
–	Head, thorax and abdomen mostly yellow with extensive reticulated brown patterning; mesoscutum and parts of pleuron white; extent of forewing markings highly variable, from relatively few markings (Fig. 3A) to more extensive, but rarely concentrated in any region of wing, apical streak lacking, hind wing unmarked except for pterostigma, pterostigmal markings relatively small	***Gryposmylus pubicosta* (Walker)**

**Figure 3.**
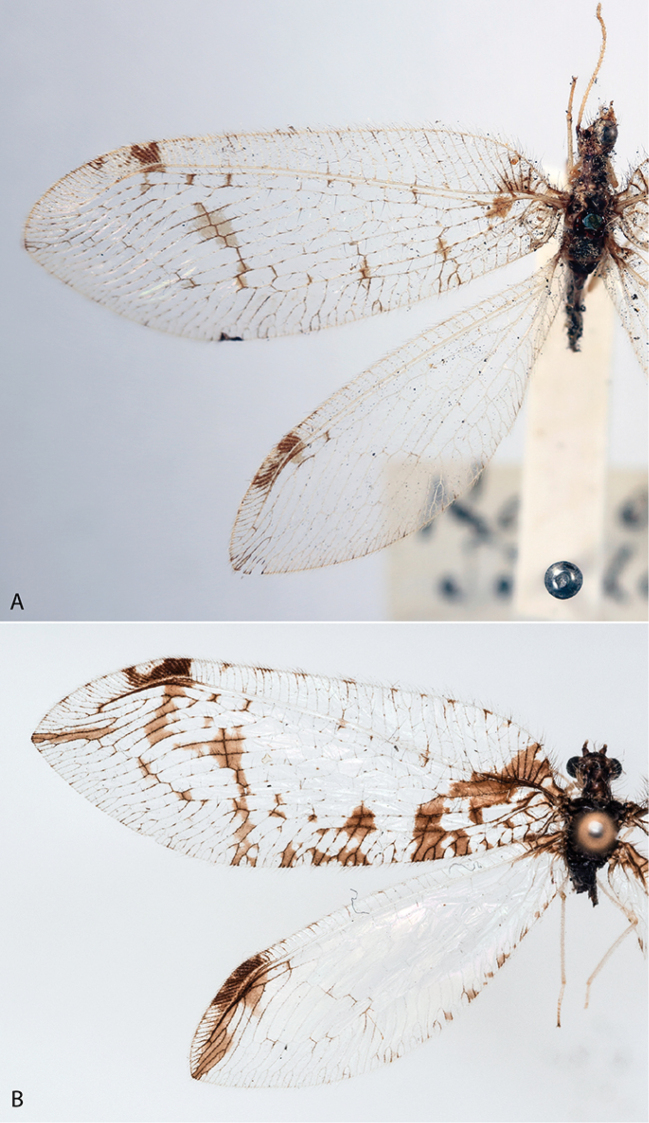
*Gryposmylus* spp.: **A** *Gryposmylus pubicosta* (Walker) (pale form) (Forewing length 16.5 mm) **B** *Gryposmylus pennyi* sp. n. (Forewing length 16.0 mm).

### Gryposmylus pubicosta

Taxon classificationAnimaliaNeuropteraOsmylidae

Walker

Figs 1 , 3A

Chrysopa pubicosta Walker 1860: 183
Gryposmylus pubicosta (Walker) – Krüger 1913: 32; Carpenter 1943: 755 [text figure 1].

#### Material examined.

**Lectotype** [sex not determined]. **INDIA**: “Hindostan” (Natural History Museum, London). Herein designated.

#### Other material examined.

**INDIA**: Himchal Pradesh Prov.: male, Kano, S[h]imla, McLachlan Collection, B.M. 1938- 674 (Natural History Museum, London); Uttarakhand Prov.: female, “Masuri” [Mussoorie], 7000 feet, 18.vi.1868, Lang McLachlan Collection, B.M. 1938- 674 (Natural History Museum, London). **MALAYSIA**: Sabah (Borneo): female, Crocker Range National Park, HQ Station Road, 9.viii.2003, Whiting, Svensen, Bybee (California State Collection of Arthropods); 2 females, Penampang Distr., Crocker Range Gunung Alab, 1660 m, 5°48'47"N 116°20'16"E [5°48.78', 116°20.26'] S. Gaimari, M. Hauser, 16–18.x.2011, ex. Mercury vapour light (California State Collection of Arthropods).

#### Diagnosis.

Head and body largely yellow with brown reticulated markings; mesoscutum and parts of pleuron white; forewing markings mottled, highly variable; hind wing unmarked except region immediately around pterostigma.

#### Redescription.

Forewing length: 21–22.0 mm; hind wing length: 16–17.5 mm. *Head*. Dark yellow with brown and white markings; palpi dark yellow; frons with dark, subtriangular marking below antennal socket, clypeus often with smaller marking laterally; dark genal mark small or large; vertex dark yellow with white area laterally, ocelli pale, surrounded with dark marking medially, dark vertex marking extending posteriorly as dark stripe from lateral ocellus; dark marking on gena along posterior margin of eye; scape dark brown, dark yellow on anterior surface; pedicel dark brown-black; flagellum dark yellow except for black basal three flagellomeres. *Thorax*. Prothorax dark yellow with black and white markings along lateral margins, setae relatively elongate, especially along lateral edge; mesothorax dark yellow with extensive dark brown-black markings, anteriorly with a dark spot and laterally with radiating pattern of brown streaks, a tuft of elongate dark setae is present anteriorly on the mesoscutum; mesoscutellum black laterally and anteriorly, vivid yellow-white posteriorly; metathorax dark yellow with dark brown spot medially, metascutellum black laterally, yellow-white posteriorly; pleuron dark yellow with broken white stripes; legs dark yellow, setal pale; claws brown. *Wings*. Rounded, venation brown with elongate setae on all veins on both surfaces of wings; wings hyaline with brown markings; extent of forewing markings highly variable, ranging from few markings to extensive markings in basal half of wing (Fig. 1), consistent markings in forewing of all specimens include: dark markings at base of costal area, at origin of M from R, crossveins 2–3cua-cup, distal crossvein ma-mp and extending along inner gradate series, distal crossveins r-rs and pterostigma; hind wing largely hyaline except for dark markings in pterostigma and distal crossveins r-rs, hind wing venation pale except for wing apex. *Abdomen*. Pale to dark yellow on all segments with dark brown reticulate pattern on tergites 1–8 and sternites 1–5; pale erect setae sparsely distributed on all segments. *Male genitalia*. Tergite 8 and sternite 8 quadrangular, sparsely distributed setae on sclerites and intersegmental membrane; tergite 9 relatively narrow, extending ventrally below level of ectoproct; sternite 9 subtriangular, fused partially to gonarcus laterally; ectoproct rounded with thickened area along posterolateral margin, callus cercus relatively large with *ca.* 45 setae; genitalia typical for subfamily, gonarcus as narrow arch medially, narrow entoprocessus extending posteriorly, curved dorsally and spatulate distally; gonarcus extending anteriorly as non-articulated rod-shaped apodemes (=baculum), gonarcus fused laterally to sternite 9 at junction of entoprocessus and gonarcus anterior apodeme; parameres narrow, arch-shaped with medial thickening; mediuncus curved with paired-flanges, connected membranously to medial arch of gonarcus. *Female genitalia*. Tergite 8 large and subquadrate, sternite 8 as small and knob-like process, directed posteriorly, adjacent to tergite 9; tergite 9 narrow, extending ventrally to articulate with gonopophysis 9 + gonocoxite 9 (=gonapophysis lateralis); gonopophyses 9 and gonocoxite 9 closely associated; gonocoxite 9 elongate with a dark longitudinal band laterally, distally articulated with a relatively long stylus (=gonostylus 9); ectoproct rounded, callus cercus relatively large; spermathecae folded medially, expanded basally and connecting with a very long coiled spermathecal duct.

#### Comments.

The specific type locality for this species is listed as “Hindostan” by Walker (1860), which is a common geographic term for the entire northwestern portion of India. The McLachlan collection, now in the Natural History Museum collection, contains multiple specimens of *Gryposmylus pubicosta*, presumably identified by Walker. Walker (1860) does not indicate that the description is based on a series of specimens, and the measurements provided in the description suggest that it was based on a single specimen. Moreover, at least one specimen in the McLachlan collection was collected in 1868, years after the original description of the species was published. Consequently, we do not consider these additional specimens as part of the syntype series but herein designate a Lectotype to clarify the status of this species.

This species is variable in the extent of body and wing markings, with some species being very pale with few wing markings (Fig. 3A) to others being very dark with extensive wing markings (Fig. 1). The male and female genitalia are very similar between both species in the genus.

### Gryposmylus pennyi sp. n.

Taxon classificationAnimaliaNeuropteraOsmylidae

http://zoobank.org/4E470ED2-B2DF-4D17-B5B1-F71AAFBDA440

**Figure 4.**
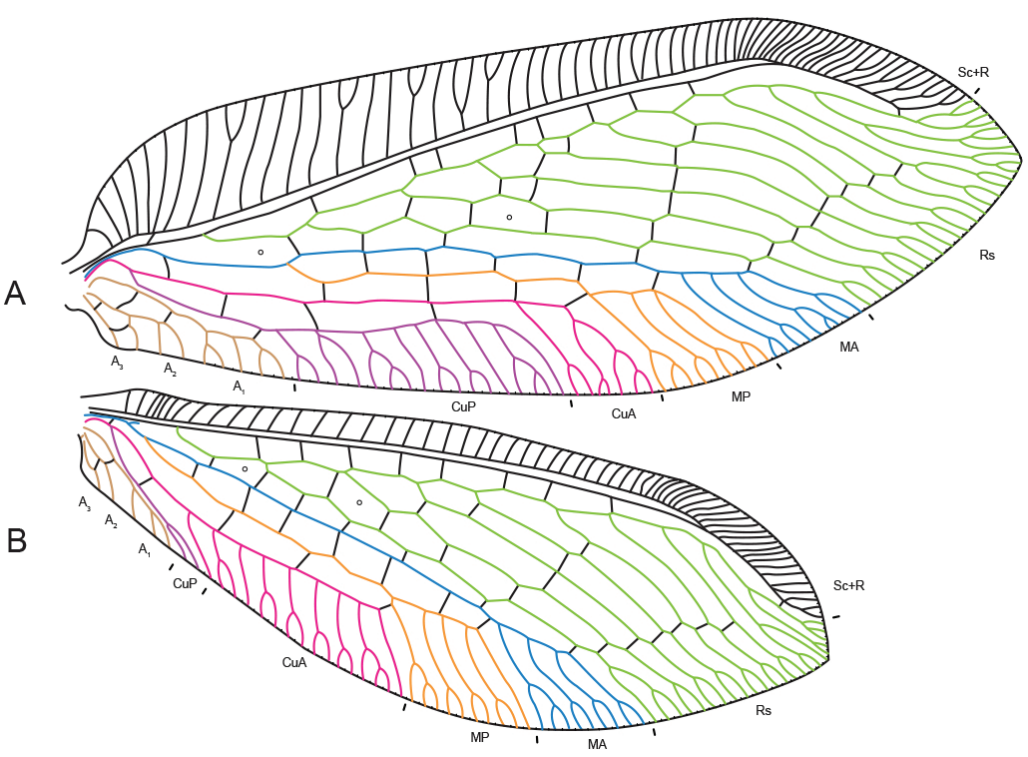
Wing venation of *Gryposmylus pennyi* sp. n.: **A** forewing **B** hind wing. Major wing veins are colour coded.

**Figure 5.**
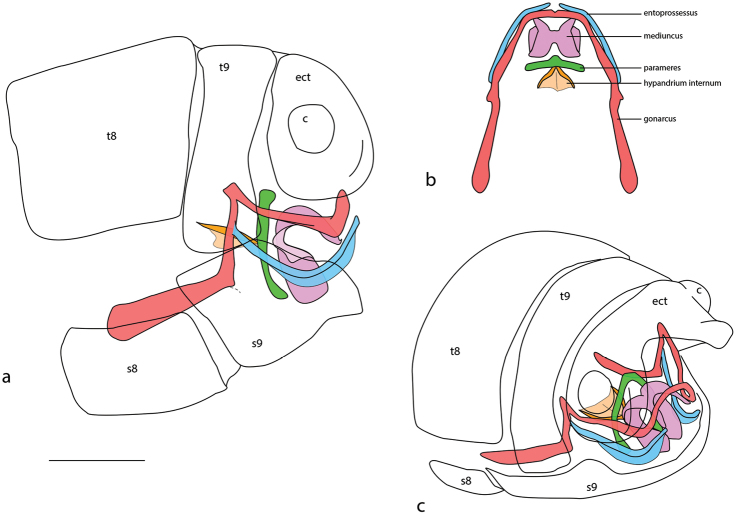
Male genitalia of *Gryposmylus pennyi* sp. n.: **A** lateral view **B** dorsal view **C** oblique view. Colour key: gonarcus (red), ectoprocessus (blue), mediuncus (purple), parameres (green), hypandrium internum (orange). Abbreviations: *t8*, tergite 8; *s8*, sternite 8; *t9*, tergite 9; *s9*, sternite 9; *ect*, ectoproct; *c*, callus cercus. Scale bar: 0.2 mm.

**Figure 6.**
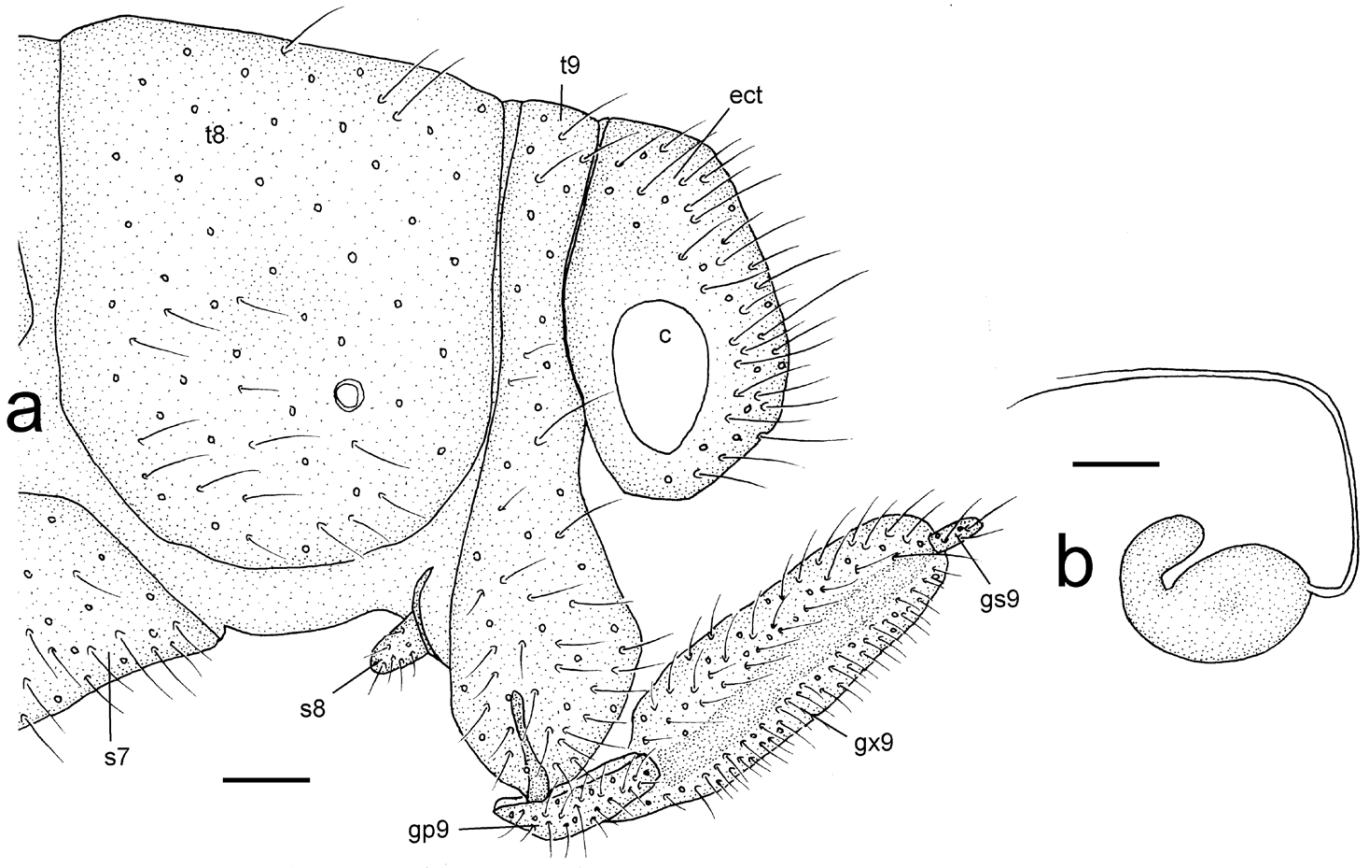
Female genitalia of *Gryposmylus pennyi* sp. n.: Additional abbreviations: *gx9*, gonocoxite 9; *gp9*, gonopophysis 9; *gs9*, gonostylus 9. Scale bars: 0.2 mm.

Figs 2 , 3B , 4 , 5 , 6

#### Material examined.

**Holotype** male. **VIETNAM**: Ninh Binh Prov.: Cuc Phueng National Park, 390m, 20°21'03"N. 105°35'36"E [20°21.05', 105°35.6'], S.D. Gaimari, M. Hauser, Pham H.T., 26.iii.2012, ex. Mercury vapour light (California State Collection of Arthropods).

**Paratype** female. **CHINA**: Yunnan Prov.: Mengla, Wangtianshu, 4.V.2005, Xiaoshuan Bai (China Agricultural University Collection).

#### Diagnosis.

Head and body largely black with dark brown markings; forewing markings with distinct dark pattern, especially basally, and elongate band apically; hind wing with markings along posterior margin and in wing apex.

#### Description.

Forewing length: 16.0–16.5 mm; hind wing length: 13.0–13.5 mm. *Head*. Predominantly black; frons cream-white with black opposing chevrons; clypeus with two black spots; gena with black spot; palpi white with dark bands on each segment; vertex black with lateral eye margin and ocelli white; antennal scape black, white on anterior surface; pedicel black; flagellum cream-white with basal flagellomere black. *Thorax*. Prothorax slightly narrowed anteriorly, predominantly black, white laterally and with three white spots along posterior margin; posterior intersegmental membrane white; prothoracic pile erect and a mixture of black and white setae; mesoscutum and metascutum black; pleuron with white and black longitudinal stripes, legs white, tibiae dark brown basally and setae on tibiae and tarsi yellowish. *Wings* (Figs 3B, 4). Forewing costal area broad with crossveins mostly simple, admixed with some forked veins (variable between wings and individuals); wing venation brown with elongate setae on all veins on both surfaces of wings; wings hyaline with extensive dark brown markings arranged in a broad sigmoid pattern (Fig. 2), extensive markings in posterior region of forewing, along both gradate series and apically along distal Rs veins; pterostigma very dark; hind wing mostly hyaline, venation pale; dark markings and venation at wing base, along posterior margin gradate series and from pterostigma to wing apex. *Abdomen*. Uniformly black, with dark brown markings. *Male genitalia* (Fig. 5). Tergite 8 and sternite 8 quadrangular, sparsely distributed setae on sclerites and intersegmental membrane; tergite 9 relatively narrow, extending ventrally below level of ectoproct; sternite 9 subtriangular, fused partially to gonarcus laterally; ectoproct rounded with thickened area along posterolateral margin, callus cercus relatively large with *ca.* 45 setae; gonarcus as narrow arch medially, narrow entoprocessus extending posteriorly, reflexed dorsally and spatulate distally; gonarcus extending anteriorly as non-articulated rod-shaped apodemes (=baculum), gonarcus fused laterally to sternite 9 at junction of entoprocessus and gonarcus anterior apodeme; parameres narrow, arch-shaped with medial thickening dorsally; mediuncus curved with paired-flanges, connected membranously to medial arch of gonarcus. *Female genitalia* (Fig. 6). Tergite 8 large and subquadrate, sternite 8 as small and knob-like process, directed posteriorly, adjacent to tergite 9; tergite 9 narrow, extending ventrally to articulate with gonopophysis 9 + gonocoxite 9 (=gonapophysis lateralis); gonopophyses 9 and gonocoxite 9 closely associated; gonocoxite 9 elongate with a dark longitudinal band laterally, distally articulated with a relatively long stylus (=gonostylus 9); ectoproct rounded, callus cercus relatively large; spermathecae folded medially, expanded basally and connecting with a very long coiled spermathecal duct.

#### Comments.

**Figure 7.**
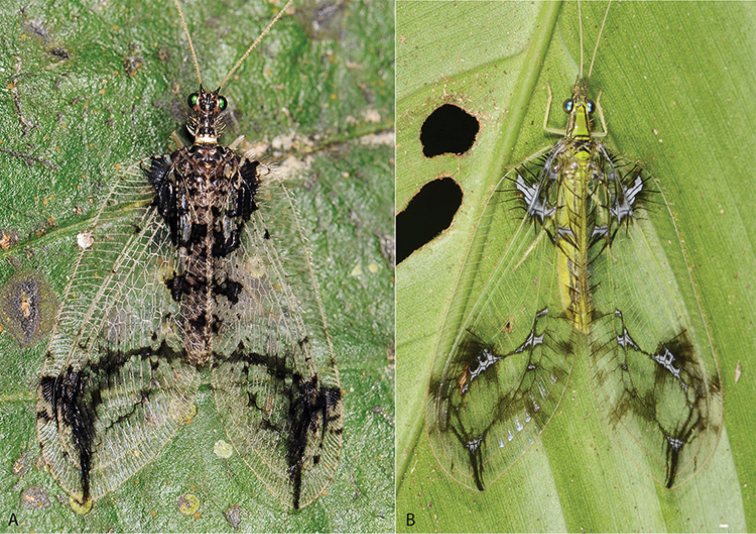
Comparison of *Gryposmylus pennyi* sp. n. (**A**) (Oriental) and *Vieira leschenaulti* Navás (**B**) (Chrysopidae) (Neotropical) (photograph credits: **A** Stephen D. Gaimari **B** Arthur Anker).

*Gryposmylus pennyi* sp. n. is distributed in northern Vietnam and adjoining southern China. A specimen was also recently photographed from Sabah, Malaysia, with the image posted on social media website ‘Facebook’; the specimen was identified but it was not collected. *Gryposmylus pennyi* sp. n. has distinctive wing markings (Fig. 7A), which show a peculiar similarity to those wing markings of an unrelated chrysopid, *Vieira leschenaulti* from the Amazon region of South America (Fig. 7B). This is a dramatic example of convergent wing patterning in distantly related lacewings, presumably associated with disruptive camouflage patterning to break up the outline of the individual as it sits on the underside of leaves in dense forested habitats.

#### Etymology.

We have the great honour of naming this species after the Late Norman Penny (1946–2016). Norm was a wonderful colleague and excellent researcher of Neuroptera, with numerous publications on various lacewing families, especially on New World Chrysopidae.

## Supplementary Material

XML Treatment for Gryposmylus

XML Treatment for Gryposmylus pubicosta

XML Treatment for Gryposmylus pennyi
